# Spt2p Defines a New Transcription-Dependent Gross Chromosomal Rearrangement Pathway

**DOI:** 10.1371/journal.pgen.1000290

**Published:** 2008-12-05

**Authors:** Nilabja Sikdar, Soma Banerjee, Han Zhang, Stephanie Smith, Kyungjae Myung

**Affiliations:** Genome Instability Section, Genetics and Molecular Biology Branch, National Human Genome Research Institute, National Institutes of Health, Bethesda, Maryland, United States of America; Fred Hutchinson Cancer Research Center, United States of America

## Abstract

Large numbers of gross chromosomal rearrangements (GCRs) are frequently observed in many cancers. High mobility group 1 (HMG1) protein is a non-histone DNA-binding protein and is highly expressed in different types of tumors. The high expression of HMG1 could alter DNA structure resulting in GCRs. Spt2p is a non-histone DNA binding protein in *Saccharomyces cerevisiae* and shares homology with mammalian HMG1 protein. We found that Spt2p overexpression enhances GCRs dependent on proteins for transcription elongation and polyadenylation. Excess Spt2p increases the number of cells in S phase and the amount of single-stranded DNA (ssDNA) that might be susceptible to cause DNA damage and GCR. Consistently, RNase H expression, which reduces levels of ssDNA, decreased GCRs in cells expressing high level of Spt2p. Lastly, high transcription in the chromosome V, the location at which GCR is monitored, also enhanced GCR formation. We propose a new pathway for GCR where DNA intermediates formed during transcription can lead to genomic instability.

## Author Summary

Transmitting genetic information without creating deleterious genetic alternations is one of the cell's most important tasks. When cells cannot repair DNA damage properly, it leads to genomic instability and results in genetic disorders, including cancer. Many studies, including ours, have started to uncover pathways suppressing one type of genomic instability, gross chromosomal rearrangement (GCR). However, the pathogenic mechanism to promote GCR that could mimic the hyper-activation of oncogenes during tumorigenesis is not clearly understood. The high expression of HMG1 has been documented many times as a putative oncogene. Therefore, we tested whether high expression of its yeast homologue, Spt2p, could induce pathogenic effect including GCR formation. Excess Spt2p expression indeed induced GCR formation dependent on its role in transcription elongation and polyadenylation. Further studies to find mechanisms resided in GCR formation by Spt2p revealed that excess Spt2p increased single-stranded DNA to produce GCR. Our studies provide a mechanistic bridge between transcription and genomic instability.

## Introduction

Maintaining genomic stability is crucial for cell survival and normal cell growth. Different genetic disorders, including cancers, display different forms of genomic instabilities. There is growing evidence supporting the hypothesis that gross chromosomal rearrangements (GCRs) found in different cancers are caused by the pre-acquisition of mutator mutations [1]–[4]. Identification of such mutator mutations could help to identify more genes participating in carcinogenesis.

Multiple mutator mutations that facilitate GCRs were identified by using the yeast *Saccharomyces cerevisiae* as a model system [2], [5]–[8]. There are multiple pathways for the suppression of genomic instability. The importance of these pathways in human cancer development has been uncovered by observations of mutations in their human homologous genes in many cancers or cells from cancer pre-disposed syndrome patients [1],[2],[4],[9].

Chromatin structure is important for almost all DNA metabolism including replication, transcription, recombination, and repair. Nucleosome, a basic unit of chromatin is composed of 146 base pairs of DNA wrapped with octameric histones [10]. Other non-histone DNA binding proteins participate in the structure of chromatin [11]. Spt2p, also known as Sin1p is a non-histone DNA binding protein and was first identified by mutations suppressing Ty and *Δ* insertion mutations in the *HIS4* gene in *Saccharomyces cerevisiae* [12]. In addition, the *spt2Δ* mutation suppresses the abnormal initiation of transcription conferred by mutations that cause defects in Swip/Snfp [13] or in the SAGA complex [14],[15] as well as by the *rpb1Δ* mutation that shortens the Rpb1p carboxyl-terminal domain [16].

The synthetic lethal interactions between *spt2Δ* and *cdc73Δ*, a member of the PAF complex, which accompanies RNA polymerase II during elongation and has an important function in polyadenylation, suggested that Spt2p could function in transcription elongation and polyadenylation [17],[18]. In addition, the functional interaction between Spt2p and Hpr1p further supported the putative role of Spt2p in transcription elongation and polyadenylation because Hpr1p is part of THO complex as well as Fir1p that is a positive regulator of RNA polyadenylation [17],[19]. Recent molecular evidence including chromatin immunoprecipitation data and the effect on polyadenylation of the *spt2Δ* mutation confirmed that Spt2p indeed functions in both transcription elongation and polyadenylation [17],[20].

In addition to its role in transcription, the *spt2Δ* mutation enhances recombination where transcription is active [17] and causes defects in chromosome segregation [21]. These data strongly suggest that Spt2p has a role in maintaining general genomic integrity, presumably where transcription is active.

Spt2p has two domains that have high homology to the high mobility group 1 (HMG1) protein in higher eukaryotes, as well as an acidic domain and a C-terminal polar helical domain [21]–[23]. Three of these domains can bind four-way junction DNA. Its DNA binding activity seems to induce specific changes in chromatin structure, thereby allowing the assembly of proteins involved in transcription and recombination [23].

In the present study, we demonstrate that excess Spt2p induces a high degree of GCR formation in *Saccharomyces cerevisiae*. The C-terminal polar helical domain (amino acids 303 to 333), which is required for DNA binding is necessary and sufficient for enhancing GCR formation. GCRs enhanced by excess Spt2p were due to an increase of single stranded DNA (ssDNA), presumably through the collision of transcription-dependent R-loops and replication forks. These findings demonstrate that defects in tight regulation between replication and transcription could lead genomic instability.

## Results

### Excess Spt2p Enhances GCR Formation

**Figure 1:**
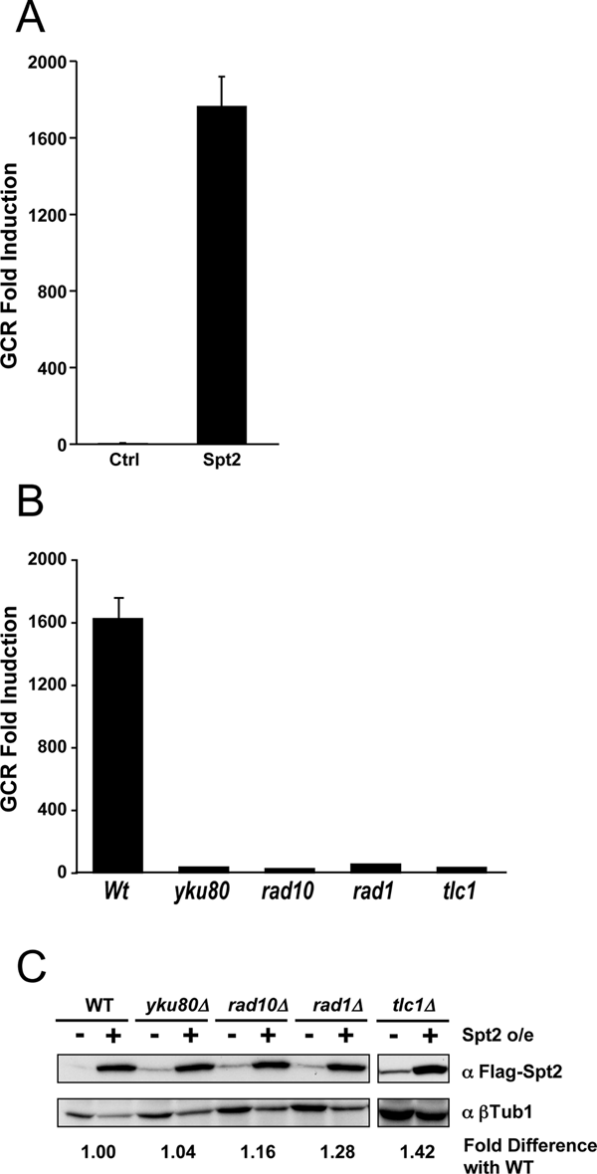
Excess Spt2p enhances GCR formation in yKu80p, Rad1p-Rad10p, and Telomerase dependent manner. A) The two-hour induction of Spt2p enhances the GCR rate as compared to the control that carried the plasmid backbone only. B) The GCR produced by excess Spt2p was significantly reduced by the mutation in *yKU80, RAD10, RAD1,* or *TLC1* genes. C) Western blot analysis of FLAG tagged Spt2p demonstrates that *yku80, rad10, rad1,* or *tlc1* mutation did not cause significant change in expression levels of Spt2p. The intensity of each band from Spt2p was divided by the intensity of band from tubulin control. The induction value was calculated by dividing the number after the galactose induction (+) with the number before the galactose induction (−). The fold difference with wild type was calculated by setting the induction value of wild type to 1.

Structural changes of chromosomes induced by overexpression of DNA binding proteins alter multiple DNA metabolisms including replication, repair, and transcription. Such changes in chromosome might lead to GCR. Dramatic increase of HMG1 expression has been documented in many tumors [24]–[26]. Yeast Spt2p has an HMG1-like motif and functions to change structure of chromosomes that affects transcription presumably through its DNA binding activity [15],[17],[20],[23]. We hypothesized that Spt2p overexpression could lead to GCR. To test this hypothesis, we overexpressed Spt2p for two hours under a galactose-inducible promoter. High expression of Spt2p enhanced GCR up to 1,600 fold compared to normal level of expression even without treatment with DNA damaging agents (Figure 1A). To determine whether the level of Spt2p expression affects GCR formation, the GCR frequencies were measured after inducing Spt2p expression for different lengths of time. A slight increase in Spt2p expression after 30 minutes was enough to increase GCR formation (Figure S1A). The maximum increase in GCRs was achieved after two hours of induction and started to decrease after four hours. When we chronically overexpressed Spt2p, cells did not grow well (Figure S1B). Therefore, the decrease in GCRs after four hours seems to be due to growth defects caused by excess Spt2p.

The rearrangement structures from sixty independent clones containing GCRs induced by excess Spt2p were all broken chromosomes healed by the addition of telomere sequence through *de novo* telomere addition, a class of GCR known as *de novo* telomere addition. Consistent with this observation, mutations in *yKU80, RAD10, RAD1*, or *TLC1* that are required for *de novo* telomere addition almost completely abolished GCRs caused by excess Spt2p (Figure 1B). This reduction in GCRs is unlikely due to a reduction in Spt2p expression, as levels of this protein were similar across all strains (Figure 1C).

**Figure 2:**
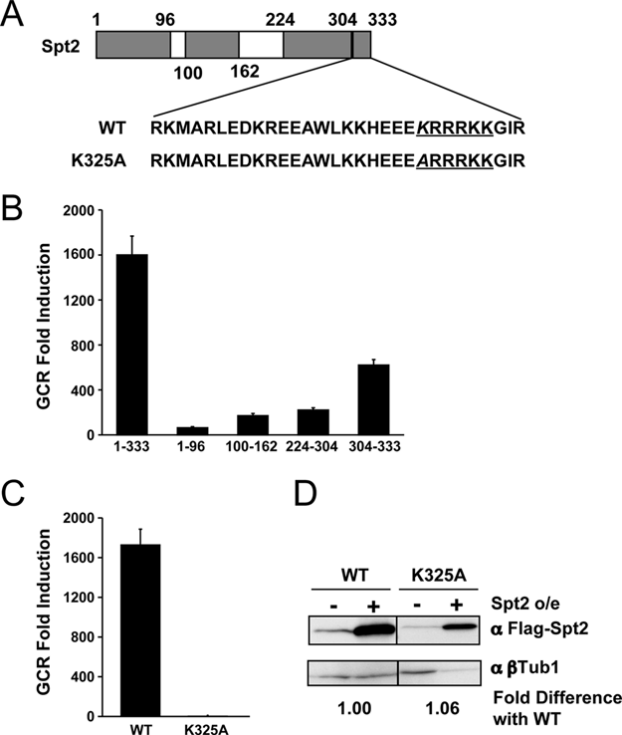
The DNA binding domain of Spt2p is important for GCR enhancement. A) Spt2p has four different domains important for DNA binding. The C-terminal domain has a lysine residue (italic letter). The K325A point mutation is located near the C-terminus of Spt2p where there are large numbers of positive charged amino acids that are underlined in the figure. B) Overexpression of different domains of Spt2p enhanced GCR formation. C) The K325A point mutation completely blocked the increased GCR produced by excess Spt2p. D) Western blot analysis confirmed that the K325A point mutation did not affect the expression or the stability of Spt2p. α represents antibody. β-tubulin1 was used as a control. The fold difference with wild type was calculated as described in Figure 1 legend.

Spt2p has two mammalian HMG1-like domains in its N terminal half and two C-terminal acidic domains, which are often found in HMG-like proteins (Figure 2A). Because all four domains have been shown to bind DNA [23] and thus potentially affect GCR formation, we cloned each domain individually and overexpressed them in the same manner as the wild type protein, and monitored GCR frequencies. Overexpression of each domain enhanced GCR in different extent. The highest enhancement was observed when the C-terminal thirty amino acids were overexpressed (Figure 2B). The last thirty amino acids of Spt2p have a cluster of positively charged amino acids that is important for the binding of Spt2p to four-way junction DNA [23] and suppression of a *swi1* phenotype [27],[28]. A single amino acid substitution at Lysine 325 to Arginine completely abolished the binding activity of Spt2p to four-way function DNA and the ability of Spt2p to suppress *swi1* phenotype. To test whether DNA binding of Spt2p is important for GCR formation, we overexpressed full length Spt2p with the K325R mutation. Although the mutant protein was expressed at a level similar to wild type, the overexpression of K325R Spt2p mutant protein did not cause any GCR formation (Figure 2C and D). Therefore, the GCR formation enhanced by excess Spt2p requires the C-terminal DNA-binding domain. The lack of GCR promoting activity of the full length Spt2p with the K324R mutation even though it has other domains that could enhance GCR separately (Figure 2B) could be due to structural differences.

### GCR Formation by Excess Spt2p Depends on Bur1p/Bur2p Kinase and Rad6p/Bre1p

**Figure 3:**
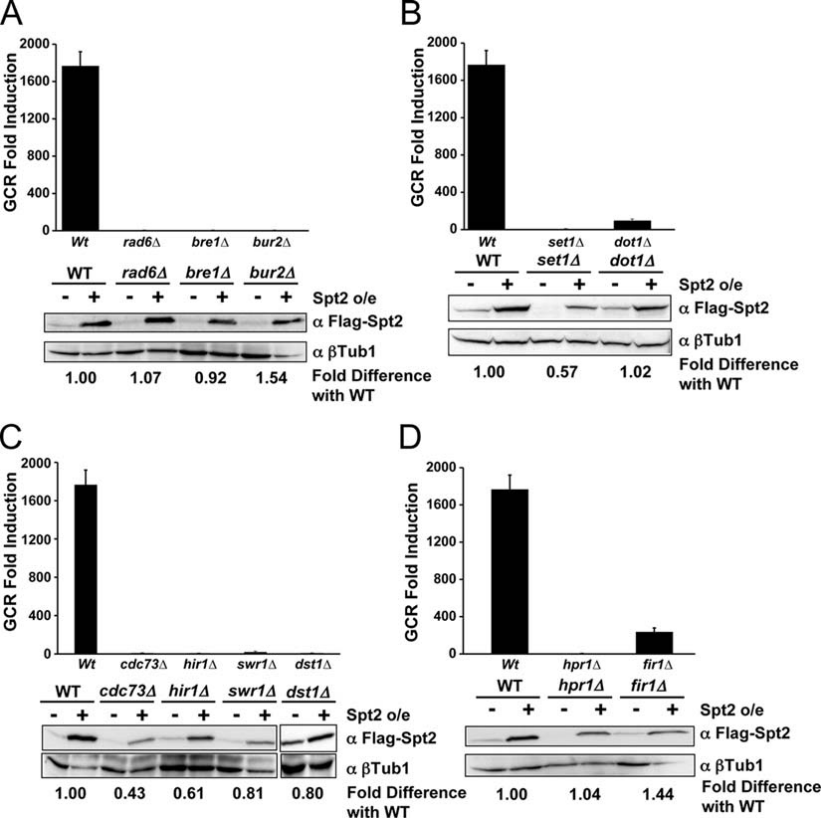
Mutations in genes functioning in transcription elongation and polyadenylation abolished GCR produced by excess Spt2p. A) Inactivation of Bur2p, Rad6p, or Bre1p blocked GCR produced by excess Spt2p. B) Mutation in either *SET1* or *DOT1* reduced the GCR formation by excess Spt2p. C) Proteins participating in transcription elongation including Cdc73p in PAF complex, Hir1p, Swr1p, and Dst1p are required for GCR caused by excess Spt2p. D) Mutation in *HPR1* or *FIR1* abolished GCR caused by excess Spt2p. The bottom panel of each section shows the expression level of Spt2p in strain backgrounds used in the study. α represents antibody. β-tubulin was used as a control. The fold difference with wild type was calculated as described in Figure 1 legend.

To investigate whether GCR enhancement by excess Spt2p has any genetic interaction with known GCR pathways, the *spt2Δ* mutation was added in *mre11Δ, mec1Δ sml1Δ,* or *pif1-m2* strains and GCR rates were determined. The additional *spt2Δ* mutation did not cause any change in GCR rates compared to parental strains (data not shown). Therefore, GCR enhancement by excess Spt2p seems to be promoted by a different mechanism. Spt2p functions in transcriptional elongation and polyadenylation [17],[20],[29]. We hypothesized that GCR induced by excess Spt2p could be due to defects in transcription. To test this hypothesis, we mutated different genes functioning in elongation and polyadenylation of transcription. The Bur1p/Bur2p complex is a cyclin-dependent protein kinase involved in the regulation of transcription elongation [30]. The Bur1p/Bur2p complex phosphorylates the serine 120 of Rad6p that activates Rad6p and Bre1p to monoubiquitinate H2B for transcription elongation. Because *BUR1* is an essential gene, we deleted the *BUR2, RAD6,* and *BRE1* genes and monitored GCR formation by excess Spt2p. Although the mutation of *BUR2, RAD6,* or *BRE1* did not cause significant change of the GCR rate (Table 1), these mutations completely abolished the increase of GCRs caused by excess Spt2p (Figure 3A). Western blots showed that Spt2p expression was not affected by the *rad6Δ*, *bre1Δ*, or *bur2Δ* mutation (Figure 3A).

**Table 1:** GCR rates of various mutations affecting transcription.

Relevant Genotype	Strain Number	GCR rate (CAN^r^ -5FOA^r^)	
Wild type	RDKY3615	3.5×10^−10^ (1)	
*rad6Δ*	YKJM4415	6.1×10^−10^ (2)	
*bre1Δ*	YKJM2233	<9.9×10^−10^ (3)	
*bur2Δ*	YKJM4459	2.0×10^−9^ (5)	
*cdc73Δ*	YKJM1445	<5.8×10^−10^ (1.5)	
*hir1Δ*	YKJM4177	9.8×10^−10^ (2.8)	
*swr1Δ*	YKJM4503	7.5×10^−10^ (2.1)	
*dst1Δ*	YKJM4505	6.8×10^−10^ (2.0)	
*set1Δ*	YKJM4501	7.4×10^−10^ (2.1)	
*dot1Δ*	YKJM0934	3.5×10^−10^ (1)	
*hpr1Δ*	YKJM4563	1.8×10^−9^ (5.3)	
*fir1Δ*	YKJM4531	1.0×10^−9^ (3.0)	
*rnh1Δ*	YKJM4473	1.7×10^−9^ (4.8)	
*rnh201Δ*	YKJM4475	6.6×10^−10^ (1.9)	

All strains are isogenic with the wild type strain, RDKY3615 *[ MATa, ura3-52, leu2Δ1, trp1Δ63, his3Δ200, lys2ΔBgl, hom3-10, ade2Δ1, ade8, hxt13:: URA3]* with the exception of the indicated mutations. ( ) indicates the rate relative to wild type. < represents less than.

### GCR Formation by excess Spt2p Depends on the H3 Trimethylation by Set1p and Dot1p

BUR kinase is functionally linked to histone H2B ubiquitination and K4 trimethylation. Recently, synthetic genetic arrays and DNA microarrays demonstrated that a functional link between the BUR kinase complex and histone modification was achieved by its ability to regulate PAF recruitment selectively to genes for histone K4 trimethylation and H2B ubiquitination [30],[31]. Histone H3 is methylated at lysines 4 and 79 positions by Set1p and Dot1p, respectively, and it is dependent on a preexisting mark on the ubiquitination of K123 on H2B [32]. We hypothesized that GCR enhanced by excess Spt2p would be dependent on the methylation of Histone H3 by Set1 and Dot1. To test this hypothesis, we compared GCR frequencies upon Spt2 overexpression in *set1Δ* and *dot1Δ* strains with wild type. Consistent with our hypothesis, the *set1* and *dot1* mutations clearly reduced GCR frequencies enhanced by excess Spt2p (Figure 3B). We observed a slight reduction of Spt2p expression in the *set1Δ* strain (Figure 3B). Similar to other mutations affecting transcription, the *set1Δ* and *dot1Δ* mutations did not increase GCR rates (Table 1).

### GCR Formation by Excess Spt2p Is Dependent on PAF and HIR1/HPC Complexes

The PAF transcription elongation complex is composed of Cdc73p, Ctr9p, Leo1p, and Rtf1p [33]–[35]. Although the exact role of the PAF complex is still unclear, defects caused by the mutation of these genes clearly indicate that the PAF complex is involved in transcription elongation. Another complex known as the HIR1/HPC complex is composed of Hir1p, Hir2p, Hir3p, and Hpc and is involved in several chromatin-related processes, including regulation of histone genes, chromatin assembly, kinetochore function, and transcription elongation [17],[36]. To confirm the dependence of GCR induced by excess Spt2p on transcription elongation, GCR frequencies caused by excess Spt2p in *cdc73Δ* and *hir1Δ* strains were measured. Although there was no significant difference in GCR rates in *cdc73Δ* and *hir1Δ* strains compared to wild type (Table 1), *cdc73Δ* and *hir1Δ* mutation completely abolished the enhancement of GCR formation by excess Spt2p (Figure 3C). We observed slight reduction of Spt2p in *cdc73Δ* and *hir1Δ* strains.

Swr1p is a member of the Snf2 family ATPases. A complex containing Swr1p incorporates the histone H2A variant Htz1 into chromatin to change chromatin structure in favor of transcription [37]. To investigate whether GCR formation by excess Spt2p could be suppressed by the *swr1Δ* mutation by lowering transcription, we measured the GCR frequency in the *swr1Δ* strain upon Spt2p overexpression. Although there was no significant change in GCR rate in the *swr1Δ* strain (Table 1), the enhanced GCR caused by excess Spt2p was completely reduced by the *swr1Δ* mutation (Figure 3C). We also tested whether its inactivation of a general transcription elongation factor Dst1p could reduce GCR formation enhanced by excess Spt2p. Similar to *swr1Δ*, even though there was no significant change of GCR rate by the *dst1Δ* mutation (Table 1), the *dst1Δ* mutation completely blocked the GCR enhancement by excess Spt2p (Figure 3C). The galactose-induced Spt2p expression was not significantly affected by either the *swr1Δ* or the *dst1Δ* mutation (Figure 3C).

### GCR Enhancement by Excess Spt2p Depends on Proteins for Proper Polyadenylation of mRNA

Spt2p interacts with Fir1p, a component of the RNA cleavage/polyadenylation complex [20],[29]. Proper RNA cleavage and polyadenylation are also dependent on Hpr1p, which has been implicated in the modification of chromatin structure and in the removal of Spt2p from chromatin [19]. We hypothesized that GCR enhanced by excess Spt2p would be dependent on proper polyadenylation. To test this hypothesis, we measured GCR frequencies upon Spt2p overexpression in *hpr1Δ* and *fir1Δ* strains compared to wild type. Consistent with our hypothesis, the enhanced GCRs caused by excess Spt2p were substantially reduced by these mutations (Figure 3D) even though these mutations did not significantly affect the expression of Spt2p (Figure 3D). The *hpr1Δ* and *fir1Δ* mutations did not cause significant changes in GCR rates as compared to wild type (Table 1). Therefore, GCRs enhanced by Spt2p depend on proper transcription elongation and termination.

### Excess Spt2p Caused the Increase of RNase H Sensitive Single Stranded DNA and Stalled Cells in S Phase

**Figure 4:**
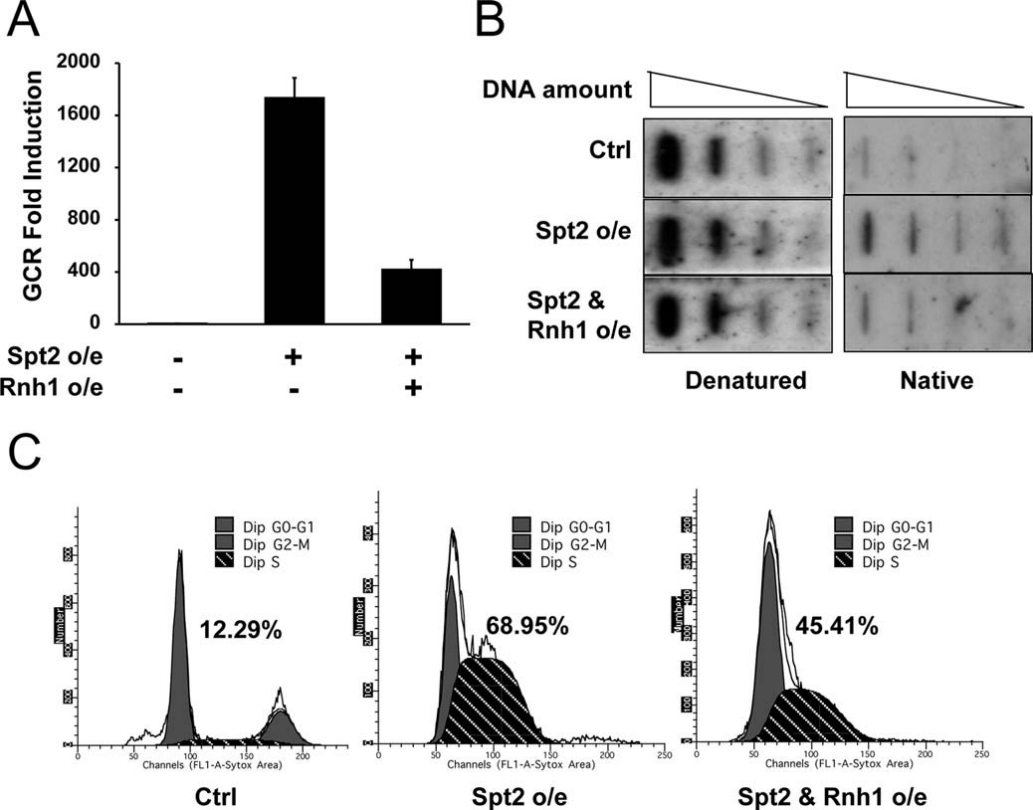
Excess Spt2 expression causes the accumulation of single stranded DNA and causes cells to arrest in S phase. A) Enhanced GCR formation by excess Spt2p was partially alleviated by RNase H expression. B) Excess Spt2p expression increased the amount of ssDNA in cells. DNA equally loaded on two membranes was subjected to hybridization with radio-labeled DNA that was PCR amplified from yeast chromosome V (31121–31859) that covers part of two ORFs, *AVT2* and *CAN1.* The denatured condition measured the quantity of DNA loaded whereas its native condition measured the amount of ssDNA. C) FACS analysis of cells carrying control plasmids, carrying Spt2p overexpression (o/e) only, and carrying both Spt2p and RNase H overexpression plasmids shows that excess Spt2p causes more cells to stay in S phase and RNase H expression decreases the S phase population.

The reduction of Spt2p-induced GCR by mutations inhibiting proper transcription suggested that abnormal transcription would produce Spt2p-induced GCRs. During transcription, the transcription machinery unwinds the DNA double helix and occupies the noncoding strand to use it as a template for transcription. In addition, transcription produces a transient DNA-RNA hybrid ranging 9 to 12 nucleotides and the coding strand becomes single stranded DNA (ssDNA). It has been shown that the hyper-recombination observed in *hpr1Δ* was due to the induction of the DNA-RNA hybrid with the R-loop formation and could be suppressed by the overexpression of RNase H [38]. We therefore hypothesized that abnormal transcription induced by excess Spt2p could increase the number of DNA-RNA hybrids and create larger ssDNA. RNase H can remove RNA from DNA-RNA hybrids. Therefore, we first tested whether removing RNA from DNA-RNA hybrids by RNase H overexpression could reduce GCRs produced by excess Spt2p. Overexpression of RNase H in addition to Spt2p substantially reduced GCR formation as compared to Spt2p overexpression alone (Figure 4A). We then compared the quantity of ssDNA when there was an excess Spt2p. Spt2p overexpression caused a high level of ssDNA that was also substantially reduced by RNase H co-overexpression (Figure 4B). Therefore, GCRs caused by excess Spt2p seemed to be produced by higher levels of ssDNA, presumably due to an abnormal transcription.

High levels of ssDNA activate a cell cycle checkpoint [39]. To test whether ssDNA created by excess Spt2p also activates cell cycle checkpoints, we investigated cell cycle profiles of cells after chronic Spt2p overexpression. In contrast to control, where there is no protein induction, Spt2p overexpression caused a significant population of cells to arrest in S phase (Figure 4C) and also arrested cell growth (Figure S1B). Consistent with a reduction of ssDNA by RNase H and Spt2p co-overexpression, the S phase population was substantially reduced by RNase H overexpression together with Spt2p overexpression. Therefore, excess Spt2p induced ssDNA presumably due to high transcription and as a result, a cell cycle checkpoint was activated. However, we could not detect Rad53 phosphorylation after the induction of Spt2p for four hours (data not shown). In addition, the *rad24Δ* mutation could not restore the growth defect of cells chronically overexpressing *Spt2p* (data not shown).

### Removal of Uracil Cenerated by Cytosine Deamination Is Important for GCRs Enhanced by excess Spt2p

**Figure 5:**
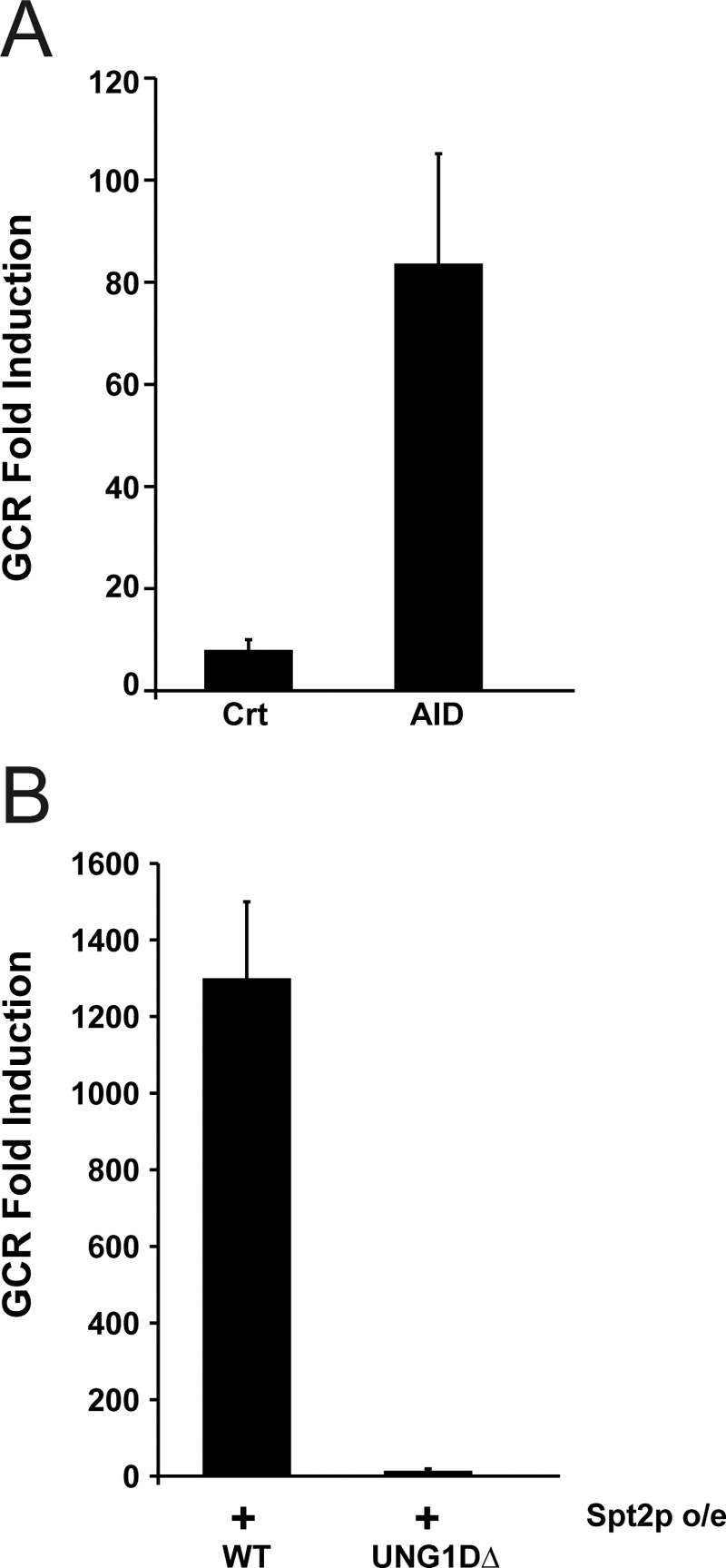
Deamination of DNA by AID increases GCR formation and the removal of Uracil from DNA is essential for GCR caused by excess Spt2p. A) Expression of human AID in yeast increased the GCR rate. B) Mutation in *UNG1* abolishes GCR caused by excess Spt2p.

Long ssDNA caused by excess Spt2p (Figure 4B) could be an easy target for multiple enzymatic reactions. Cytosines in ssDNA can be modified through deamination and changed to Uracil. Such modification by activation-induced deaminase (AID) in immunoglobulin genes causes somatic hypermutation and class switch recombination [40]. Uracil produced by deamination results in error prone hypermutation or strand breaks. We hypothesized that long ssDNA produced by excess Spt2p would be modified by AID-like enzymes in yeast to induce strand breaks for GCR formation. To test this hypothesis, we expressed the human AID enzyme in yeast and measured GCR frequency. The human AID enzyme has been shown to cause a hyper-mutation and hyper-recombination phenotype in yeast similar to in human B cells [41],[42]. Consistent with our hypothesis, the induction of human AID expression increased GCR frequency as compared to control (Figure 5A).

Ung1p, a uracil DNA glycosylase, removes uracil from DNA in yeast [43]. The removal of uracil from DNA could generate nicks in DNA. We hypothesized that strand breaks by Ung1p would be a necessary step for GCR formation by excess Spt2p. To test this hypothesis, we knocked out *UNG1* and measured GCR frequency upon Spt2p overexpression. Consistent with our hypothesis, the inactivation of Ung1p significantly reduced GCRs produced by excess Spt2p (Figure 5B). Therefore, GCRs by excess Spt2p are dependent on Ung1p that presumably creates breaks at modified uracils in ssDNA.

### High Level of Transcription Enhanced GCR Formation

**Figure 6:**
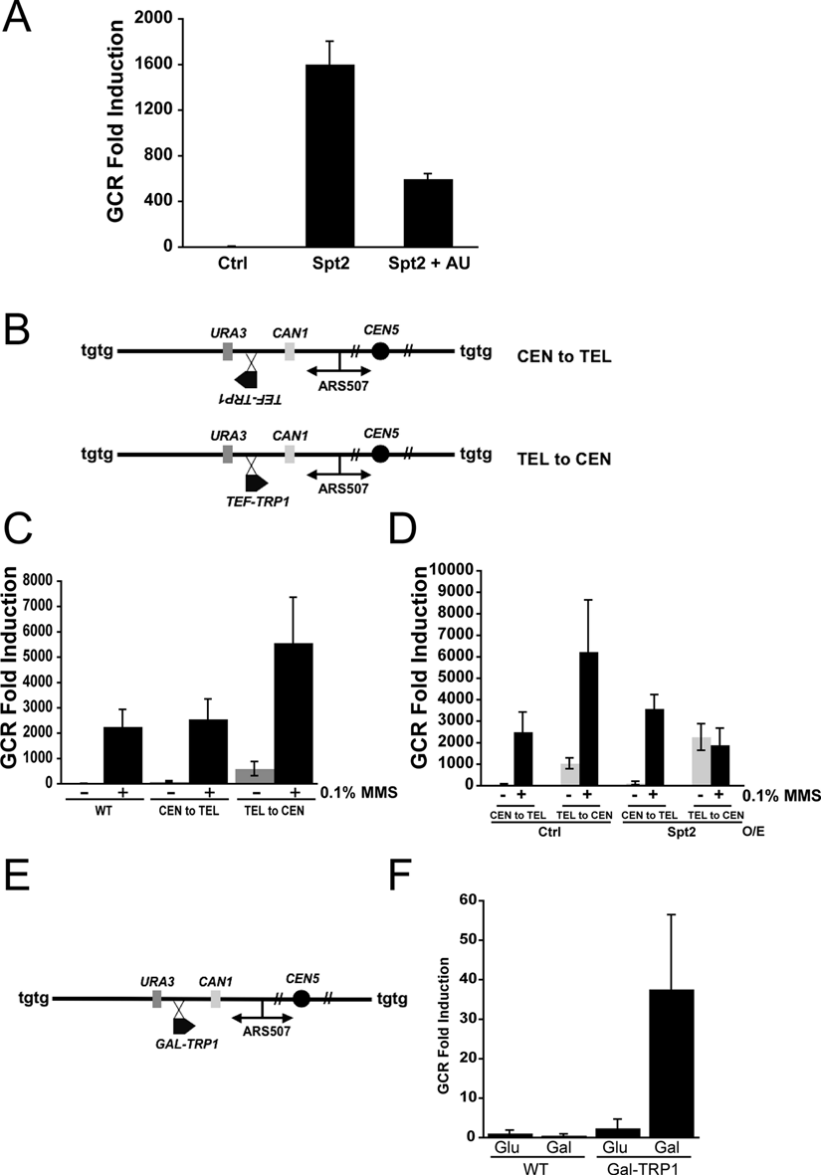
Transcription is a cause of GCR formation when transcription complexes collide with stalled replication forks. A) Treatment of 6-AU suppresses GCR caused by excess Spt2p. B) Chromosome V structures of two yeast strains; CEN to TEL chromosome V has the *TEF-TRP1* gene inserted between the *URA3* and *CAN1* genes from centromeric to telomeric direction. TEL to CEN chromosome V carries the *TEF-TRP1* gene in the same location in the opposite direction. C) TEL to CEN strain enhances GCR formation. The GCR fold induction of each strain with or without 0.1% MMS treatment is demonstrated. WT represents a strain having no *TEF-TRP1* gene. D) Excess Spt2p enhances further in GCR formation in TEL to CEN strain. GCR fold inductions from experiments were calculated by setting the GCR frequency of WT strain without MMS treatment as 1. – and + represents without and with treatment of MMS, respectively. Ctrl represents no Spt2p overexpression. E) Chromosome V structure of strain having Galactose inducible *TRP1* gene (*GAL-TRP1*) between marker genes, *CAN1* and *URA3* for GCR assay. F) Galactose driven transcription of *TRP1* gene enhanced GCR formation. WT represents a strain having no *GAL-TRP1* gene. Glu and Gal represent glucose and galactose supplied in media, respectively.

As an independent method to investigate whether transcription is a factor that enhances GCR formation, we treated yeast strain overexpressing Spt2p with 6-Azauracil (AU) and monitored the GCR formation. 6-AU is an inhibitor of enzymes involved in nucleotide biosynthesis and causes change in nucleotide pool levels. It has been shown that the treatment of 6-AU on yeast diminished transcription elongation [44]. The treatment of 6-AU significantly reduced GCRs produced by excess Spt2p (Figure 6A). Therefore, transcription elongation is an important factor for increased levels of GCR by excess Spt2p expression.

To investigate the direct involvement of transcription in GCR formation, we inserted the *TRP1* gene under the control of strong *TEF* promoter between two negative selection marker genes, *CAN1* and *URA3* for GCR assay. The *TEF-TRP1* gene was inserted in two different orientations; one transcribing the *TRP1* gene from centromeric to telomeric direction (CEN to TEL) and the other transcribing the *TRP1* gene from telomeric to centromeric direction (TEL to CEN) (Figure 6B). The GCR rate of the TEL to CEN strain was significantly higher than the rates of wild type or of the CEN to TEL strain (Figure 6C). In addition, when we measured the GCR frequencies after 0.1% MMS treatment, the TEL to CEN strain had significantly increased GCR frequency when compared to the no *TRP1* insertion (WT) or the CEN to TEL strain. Because the GCR assay marker genes seem to preferentially replicate from centromeric to telomeric direction, the collision between transcriptions and stalled forks might be the major cause of the high induction of GCR frequency in the TEL to CEN strain. Spt2p overexpression would further enhance GCR formation in these strains because it would modify the transcription rate of *TEF-TRP1.* Indeed, Spt2p overexpression further enhanced GCR formation (Figure 6D). Intriguingly, when 0.1% MMS was treated together with Spt2p overexpression, GCR frequency was reduced.

To further support that transcription caused GCR formation, we constructed another strain having a *TRP1* gene expressed under the galactose inducible promoter in the TEL to CEN direction (Figure 6E). When this strain was cultured in media having galactose that induced the expression of *TRP1* gene, GCR formation was enhanced (Figure 6F). Therefore, a high level of transcription promotes GCR formation.

## Discussion

Spt2p binds DNA and regulates transcription elongation and chromatin structure [15],[17],[20],[23]. The synthetic lethality of *spt2Δ* with other transcription elongation genes strongly suggests that Spt2p functions in transcription through its sequence non-specific DNA binding activity [17]. In addition, the absence of Spt2p caused a loss of histone H3 in transcribed regions and increased recombination between inverted repeats [17]. Therefore, Spt2p's sequence non-specific DNA binding activity seems to contribute to genomic integrity, presumably through the regulation of chromatin structure in the transcribed region.

**Figure 7:**
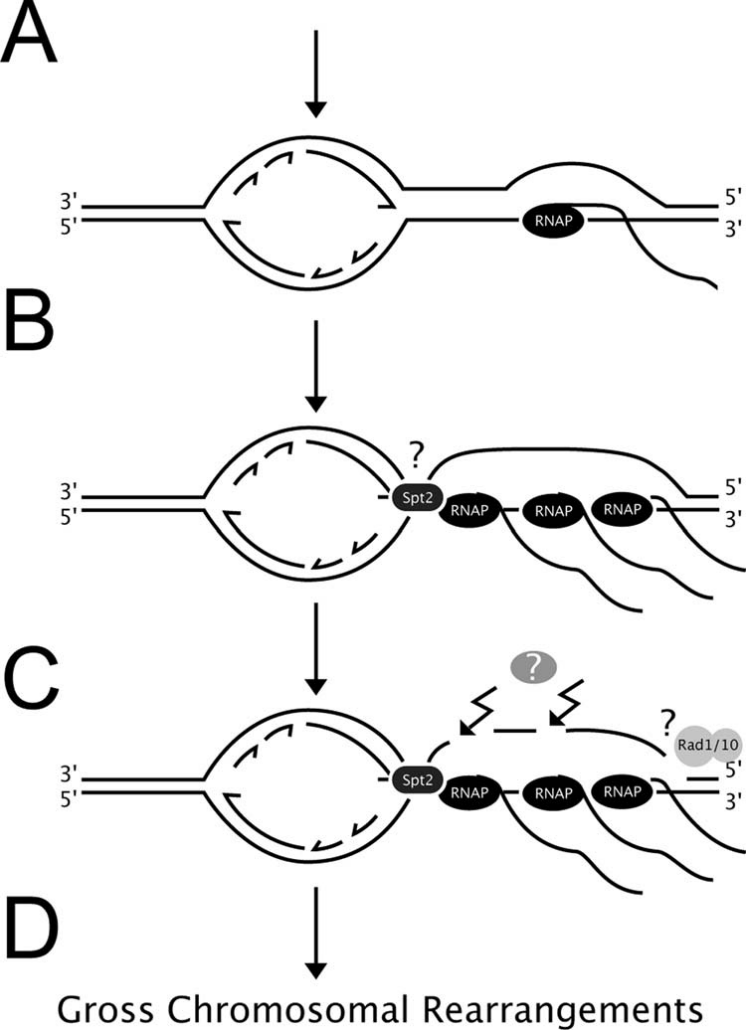
A model for GCR formation caused by excess Spt2p. A) DNA replication and transcription independently proceed to avoid unnecessary collision. A small R-loop will not trigger any specific problem. B) In the presence of excess Spt2p, the transcription rate might be enhanced and multiple RNA polymerases could occupy the transcribed strand to produce a larger R-loop. In addition, Spt2p could bind the junction between the DNA replication fork and the transcription fork. C) Some unknown AID like protein (Gray circle with ?) could introduce uracil by the deamination of cytocine in the large R loop. Uracil would enable the enzyme Ung1p to make a single strand break. Alternatively, the Rad1-Rad10 endonuclease could generate single strand break. D) Chromosomal breaks would lead to GCR formation. RNAP represent RNA polymerase.

Complete suppression of GCR caused by excess Spt2p by mutations affecting transcription (Figure 3) strongly demonstrates that excess Spt2p might alter transcription and result in GCR formation. The suppression of GCR by these mutations was specific for excess Spt2p-directed GCRs because the *fir1Δ*, *set1Δ*, or *cdc73Δ* mutation did not suppress the *mre11Δ* mutation-directed GCR formation (data not shown). It should be pointed out that a *set1Δ* or *cdc73Δ* mutation caused a slight growth defect in the *mre11Δ* strain. Transcription synergistically increases the hyper-recombinogenic effect of methyl methane-sulfonate (MMS), suggesting that transcription makes DNA more accessible to genotoxic agents [45]. Transcription also introduces topological change that could lead to transient accumulation of ssDNA. The changes in topology and chromatin structure caused by excess Spt2p could produce ssDNA because more RNA polymerase II could occupy the transcribed strands and result in the enlargement of R loops (Figure 7). In addition, excess Spt2p could bind to the collided junction between the DNA replication fork and transcription that mimics a four-way junction structure through its binding activity to four-way junction structure. The longer un-transcribed ssDNA by excess Spt2p is supported by the high-levels of ssDNA, which produced by excess Spt2p (Figure 4) and the decrease in ssDNA and GCR formation by RNase H, which removes DNA-RNA hybrids (Figure 4A and B). It has been known that ssDNA is a better substrate for many chemical reactions than double-stranded DNA [46],[47]. Long ssDNA can easily be targeted by many modifications including deamination, oxidation as well as simple breaks. Uracil introduced by the deamination of cytosine in ssDNA could be one of the intermediates for GCR formation by excess Spt2p, because expression of human AID that deaminates cytosine increased GCR formation (Figure 5A) and Ung1p, an enzyme responsible for removal of Uracil from DNA is required for GCR caused by excess Spt2p (Figure 5B). Similar to what we observed, the hyper-recombination in the *hpr1Δ* strain was caused by the increase of DNA-RNA hybrid with the R-loop formation [38].

In addition, large R loops could be mis-recognized as an intermediate in nucleotide excision repair (Figure 7). When there is DNA damage caused by ultra-violet radiation, nucleotide excision repair proteins denature damaged DNA and create bubble structure. Each end of the bubble is targeted by endonucleases to remove the damaged strand. Yeast Rad1p-Rad10p endonuclease that is homologous to human ERCC1-XPF, makes a nick in the bubble [48]. Indeed, ERCC1-XPF could cleave R loops formed in the switch regions during immunoglobulin heavy chain switch recombination in vitro [49]. Strong suppression of excess Spt2p-dependent GCR by the *rad1Δ* or the *rad10Δ* mutation (Figure 1B) suggests that large R-loops could be targeted by Rad1p-Rad10p endonuclease. However, we could not detect any significant difference in the level of overall transcription in microarray experiments (data not shown), which could be due to subtle difference in the level of transcription.

Defects in proper DNA replication seem to be a major source of spontaneous GCRs because GCRs accumulate in eukaryotes when S phase checkpoints are abrogated [50]-[52]. High levels of transcription may cause more collisions between transcription and DNA replication (Figure 7). Recombination at stalled DNA replication forks increases if there is transcription colliding with it [53]. Excess Spt2p increased the population of cells highly in S phase and RNase H could partially reverse this effect (Figure 4C). Therefore, large R loops produced by excess Spt2p could be caused mainly in S phase during DNA replication, presumably due to increased collision between transcription complexes and stalled DNA replication forks. The high increase in GCR in the TEL to CEN strain, containing the highly transcribed *TRP1* gene between two negative selection marker genes *CAN1* and *URA3* supports the collision model for GCR (Figure 6C). This model is further supported by a GCR increase observed in the *GAL-TRP1* strain only when the expression of the *TRP1* gene was induced by galactose (Figure 6F). Interestingly, the CEN to TEL strain containing the same high transcription *TRP1* gene in a reverse orientation did not cause any significant increase in GCR. It might be due to higher preference of DNA replication in this region of chromosome V from centromeric to telomeric by using ARS507 even though there are multiple late origins at the end of chromosome V. Alternatively, the TEL to CEN strain might have more susceptible chromosome structure for GCR because different orientation of *TRP1* gene could produce different chromosome structures.

The Rad5p-Rad18p dependent post-replication repair pathway suppresses GCR formation [8],[54]. In contrast to Rad18p-Rad6p that monoubiquitinates proliferating cell nuclear antigen (PCNA) and suppresses GCR formation, Bre1p-Rad6p that monoubiquitinates histone H2B, is required to promote GCR formation in the *rad5Δ, rad18Δ,* or *mec1Δ* strains [54]. In the present study, we found that GCRs produced by excess Spt2p were also suppressed by the *rad6Δ* or *bre1Δ* mutation (Figure 3A). GCRs from each individual clone carrying a GCR were all broken chromosomes healed by *de novo* telomere addition requiring telomerase and the yKu70-yKu80 heterodimer (Figure 1B). The same type of GCR was observed in *rad5Δ, rad18Δ,* or *mec1Δ* strain [8],[52]. Therefore, it is possible that certain types of GCR could be preferentially generated when DNA damage at stalled forks collide with transcription complexes. Further investigations are necessary to elucidate mechanisms. Intriguingly, Rad5p has a Swi2/Snf2 domain that has been suggested to function in altering chromatin structure. Although there is no direct evidence that yeast Rad5p functions in transcription, it might modulate transcription of genes near the stalled DNA replication forks.

The HMG1 protein is a non-histone DNA binding protein and regulates the transcription of many genes through its interaction with other proteins involved in transcription. Transcription profiling showed a dramatic increase of HMG1 expression in more than 80% of gastric cancers [24]. In addition, various cancer cells including melanoma expressed higher levels of HMG1 protein [25],[26]. Therefore, high levels of HMG1 protein seem to be closely linked to carcinogenesis. Previous studies of HMG1 overexpression in cancers mainly revealed its role in activating transcription of certain genes such as the Melanoma Inhibitory Activity (MIA) for the progression of carcinogenesis [25]. Our novel discovery demonstrating the high enhancement of GCR formation by an HMG-like protein suggests that Spt2p can add new mechanistic detail to carcinogenesis linked to transcription imbalance and genomic integrity.

## Materials and Methods

### Strains

The strains used in this study were isogenic to S288c background RDKY3615 *(MATa ura3-52 leu2Δ1 trp1Δ63 his3Δ200 lys2-Bgl hom3-10 ade2Δ1 ade8hxt13::URA3)*. All strains were generated using standard PCR-based gene disruption methods and correct gene disruptions were verified by PCR as described previously [8],[52]. The sequences of primers used to generate disruption cassettes and to confirm disruption of indicated genes are available upon request. The detailed genotypes of strains are listed in Table S1.

### General Genetic Methods

Media for the propagation of strains were as previously described [8],[52]. All *S*. *cerevisiae* strains were propagated at 30°C. Yeast transformation, yeast chromosomal DNA isolation for use as PCR template in and PCRs were performed as previously described [8],[52].

### Characterization of GCR Frequencies, Rates, and Breakpoints

400 ml of overnight cultured yeast in selective synthetic drop-out (SD) media and containing 2% glucose was inoculated into 10 ml fresh media and grown at 30°C to a cell density of 1–2×10^7^ cells/ml. Cells were washed twice with 10 ml distilled water and resuspended in10 ml of selective SD media with 2% (w/v) glycerol and 1% Succinic acid and cultured at 30°C overnight. Freshly prepared galactose was added to a final concentration of 2% to induce the expression of Spt2p. After 2 hours, cells were harvested from 1 ml of culture, resuspended in 10 ml of yeast extract-peptone media containing 2% glucose (YPD), and incubated overnight until the culture reached saturation. The cells were plated onto YPD plates and plates containing both 5-fluoroorotic acid (5-FOA) and canavanine (FC) for selection of clones with GCRs. The GCR frequency was calculated by dividing the number of colonies resistant to both drugs with actual plated cell numbers that were deduced from the number of colonies on YPD plates. Five independent cultures of each strain were used in each experiment and each experiment was performed at least twice. The average fold increases in the GCR frequency of treatment relative to that of each control were calculated. All GCR rates were determined independently by fluctuation analysis using the method of the median with at least two independent clones two or more times using 5 to 11 cultures for each clone. The average value is reported as previously described 6,55. The breakpoint spectra from mutants carrying independent rearrangements were determined and classified as described [6],[8],[55].

### Construction of Plasmids

The full-length *SPT2* gene was amplified from yeast chromosomal DNA by PCR with the primers PRKJM804 (5′ggatccGTGAAATATTTTAGTTATGAGTTTTCTTTCC3′) and PRKJM805 (5′ctcgagCAAAACATATATCAATATTCCTTAGCG3′). The sequences in lower case are additional sequences for restriction enzyme digestion for cloning purposes. The amplified *SPT2* gene was first cloned in the PCR 2.1 vector and (Invitrogen) and named pKJM371 (*SPT2*). The *SPT2* gene was sequenced to confirm that there was no mutation and then subcloned into the pYES3CT plasmid (Invitrogen), which allows the *SPT2* gene to be expressed under the GAL1 inducible promoter. This plasmid was named pKJM378 and transformed into different yeast strains for induction of Spt2 expression. As a control, the pYES3CT empty vector was transformed into the same yeast strains for comparison. The N-terminal cDNA of the *SPT2* gene encoding amino acids from 1 to 96 was PCR amplified by using PRKJM1790 (5′ccccggatccATGAGTTTTCTTTCCAAACTT3′) and PRKJM1791 (5′ ccccgcggccgcccTTAAAGGCCACCTTCATCATCGTCAT3′). The middle portion of the cDNA of the *SPT2* gene encoding amino acids from 100 to 162 was PCR amplified by using PRKJM1792 (5′ ccccggatccATGTTTAAGAGGTCTATTGGAGCA3′) and PRKJM1793 (5′ ccccgcggccgcccTTAGAAACCTGGCTTGTTAAAATGTG3′). The PCR amplification of the *SPT2* cDNA from amino acids 225 to 304 was achieved using primers PRKJM1794 (5′ ccccggatccATGAGATACCAGGATGACTATGAT3′) and PRKJM1795 (5′ ccccgcggccgcccTTATCTTGCCATTTCCTCCTCTTCC3′). The PCR amplification of the *SPT2* gene from amino acids 304 to 333 was performed using primers PRKJM1796 (5′ ccccggatccATGAGAAAAATGGCAAGGTTAGAG3′) and PRKJM1797 (5′ ccccgcggccgcTTAGCGTATGCCCTTCTTACGG3′). The *SPT2* cDNA from amino acids 1 to 303 was PCR amplified with primers PRKJM1790 (5′ccccggatccATGAGTTTTCTTTCCAAACTT3′) and PRKJM1795 (5′ ccccgcggccgcccTTATCTTGCCATTTCCTCCTCTTCC3′). The single amino acid substitution mutant Spt2p (K325A) was generated by site-directed mutagenesis with primers, PRKJM1872 (5′ AGCATGAAGAGGAGgcGAGACGCCGTAAGAA 3′) and PRKJM1873 (5′ TTCTTACGGCGTCTCgcCTCCTCTTCATGCT 3′). The lower case sequences indicate mutations incorporated to make the K325A mutation. The pKJM378 plasmid was used as a template for all PCR amplifications. All amplified PCR products were first subcloned into the PCR 2.1 vector and sequenced to confirm that there was no mutation. The plasmids carrying the *SPT2* cDNA 1–96, 100–162, 224–304, 304–333 1–303, and the K325A mutation in pCR2.1 backbone were named pKJM916, pKJM918, pKJM922 pKJM920 pKJM970 and pKJM980, respectively. All inserts were moved to the pYES3CT and named as pKJM924, pKJM926, pKJM928 pKJM930 pKJM972 and pKJM985, respectively, and were used to transform different yeast strains.

The *RNH1* gene was amplified with the primers PRKJM1891 (5′ gggaattcATGGCAAGGCAAGGGAACTTCTACGCGG) and PRKJM1892 (5′ ggctcgagTTATCGTCTAGATGCTCCTTTCTTCGCC 3′) from the yeast chromosomal DNA and subcloned into the pYX243 vector in the same manner with the construction of plasmids expressing its insert under GAL1 promoter and named pKJM1011.

### Construction of FLAG Tagged Spt2

To measure the level of the Spt2 protein expression, the *SPT2* gene was tagged at the N terminus. The FLAG tag was added into the N-terminus of the *SPT2* gene through PCR amplification of the *SPT2* gene with primers PRKJM1859 (5′ ggggatccATGGACTACAAAGACCATGACGGTGATTATAAAGATCATGACATCGATTACAAGGATGACGATGACAAGAGTTTTCTTTCCAAACTTTCCCA 3′) and PRKJM1797 (5′ ccccgcggccgcTTAGCGTATGCCCTTCTTACGG3′). The sequences in lower case are additional sequences for restriction enzyme digestion for cloning purposes. The amplified FLAG tagged *SPT2* gene was cloned in the PCR 2.1 vector and the insert was sequenced to confirm that there was no mutation. The *SPT2* gene was moved into pYES3CT and named pKJM989. Each mutation used in the study was amplified similarly by using the same primers with different templates. GCR frequencies were not affected by FLAG tag.

### FACS Analysis

To determine the cell cycles of yeast strains, FACS analysis was performed. Indicated yeast strains were grown in 2 ml of synthetic drop-out media with 2% glucose. Tryptophan or Leucine was omitted from media to support plasmids. One milliliter of the overnight cultured yeast was collected and washed three times with sterile water. Cells were resuspended in 1 ml of synthetic drop-out media with 2% galactose and allowed to grow for an additional 24 hours for induction of the Spt2 gene. Cells (0.5 ml; 1–2×10^6^) were washed and resuspended in cold 70% ethanol followed by 2 hour incubation on ice. Cells were then incubated with 0.5 ml of 1 mg/ml RNase containing 50 mM Tris HCl (pH 7.4) and 15 mM NaCl overnight at 37°C. Cells were harvested and resuspended in 0.5 ml of 50 mM Tris HCl (pH 7.4) and 50 µl of cell suspension was placed into 1 ml of SYTOX Green solution (1 µM SYTOX Green in 50 mM Tris HCl pH 7.4), was sonicated at low power, and was analyzed by standard flow cytometry methods. For this study, cells were analyzed on a FACScalibur (Becton Dickinson Immunocytometry Systems), with an argon laser tuned to 488 nm. The FL1 detector with a standard 530/30 band pass filter was used in the acquisition of SYTOX Green fluorescence and the FL3 detector with a 670 nm long pass filter was used to collect PI fluorescence.

### Western Hybridization

Cell extracts were prepared by a standard trichloroacetic acid (TCA) method. Briefly, cells were washed with 20% trichloroacetic acid and broken with glass beads. Cell extracts were collected and resuspended in 1X SDS loading buffer. Samples were boiled and centrifuged before being loaded onto a 7–12% SDS PAGE (Bio-Rad). Proteins separated by SDS PAGE were transferred to a PVDF membrane and FLAG-Spt2 was detected by standard western hybridization with an anti-FLAG HRP antibody (Sigma) and Western Blotting Detection Reagents (GE Healthcare).

### Detection of Single Stranded DNA (ssDNA)

Cells were prepared by using the same method described in FACS analysis. Chromosomal DNA was prepared using Gentra Puregene yeast chromosomal DNA isolation kit (Qiagen) following the manufacturer's protocol. The same quantity of chromosomal DNA was spotted on nitrocellulose membrane in duplicate. DNA in one membrane was denatured via incubation with the denaturation solution (0.5M NaOH, 1.5M NaCl) for 30 minutes followed by incubation with the neutralization solution (0.5M Tris-HCl, 3M NaCl, pH7.4) for thirty minutes at room temperature. After UV crosslinking of DNA, membranes were hybridized with radio-labeled DNA by random priming with Prime II (Stratagene). The DNA used for probe was PCR amplified from yeast chromosome V (31121–31859) that covers part of two ORFs, *AVT2* and *CAN1* with primers PP1-1 (5′-CCTTGGCTTCCGTCATCGGAGTCGTTATCAG-3′) and PP1-2 (5′-GCTTTGCTGCCGCCTATATCTCTATTTTCCTG-3′).

## Supporting Information

Figure S1 Excess Spt2p enhances GCR formation and causes growth arrest. A) The maximum GCR enhancement was achieved after two hours induction of Spt2p under the galactose promoter. The intensity of each band from Spt2p was divided by the intensity of band from tubulin control. The number generated from time 0 was set to 1 and the fold induction was calculated. B) Chronic expression of Spt2p causes growth arrest.
(0.39 MB TIF)

Table S1 *S. cerevisiae* strains used in this study.
(0.16 MB DOC)
