# Supplementary figures and images for: Spt2p Defines a New Transcription-Dependent Gross Chromosomal Rearrangement Pathway
Source: PLoS Genet. 2008 Dec 5;4(12):e1000290. doi: 10.1371/journal.pgen.1000290 (PMC2585797; doi:10.1371/journal.pgen.1000290)

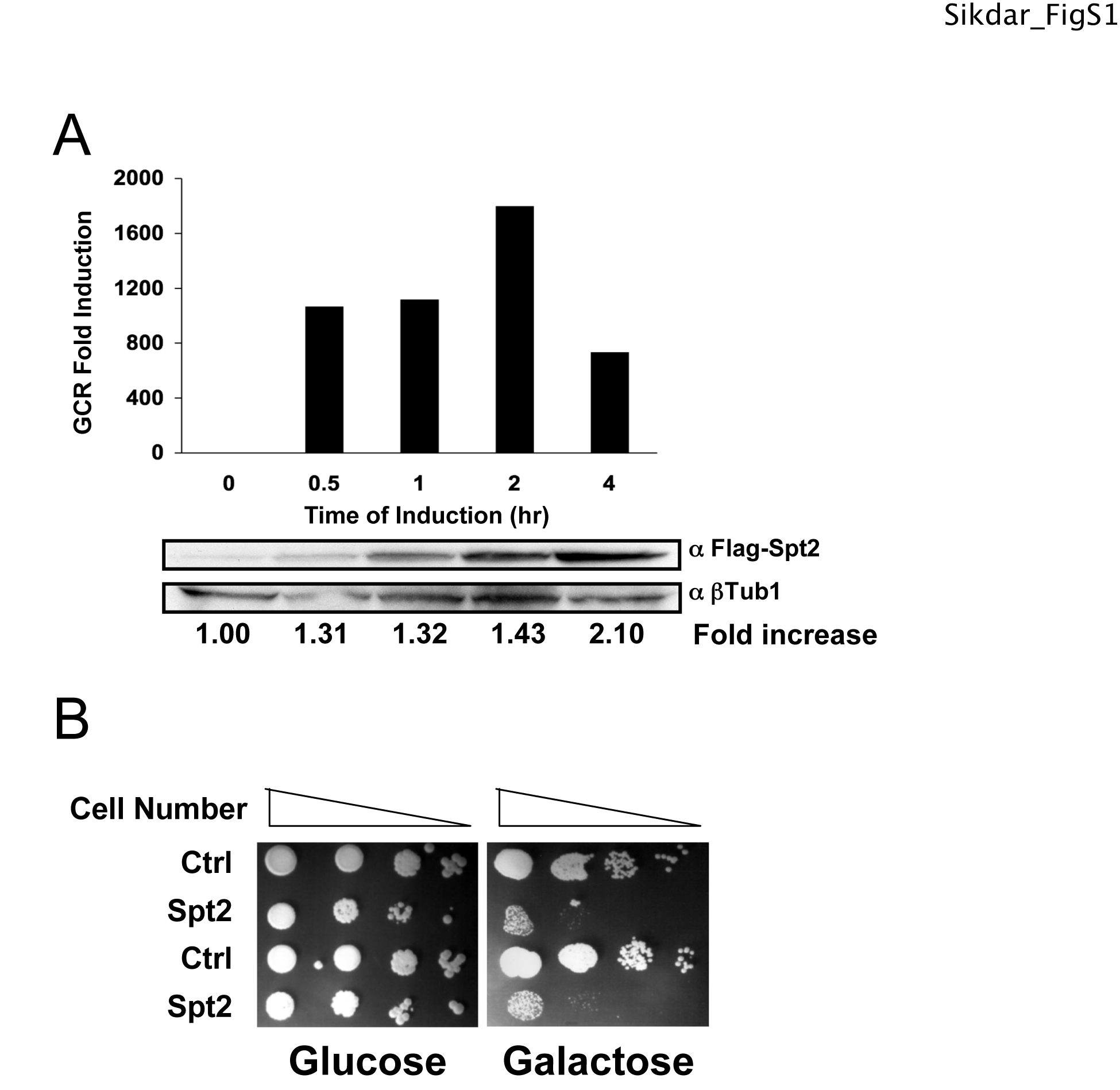

Supplement: Figure S1 — Excess Spt2p enhances GCR formation and causes growth arrest. A) The maximum GCR enhancement was achieved after two hours induction of Spt2p under the galactose promoter. The intensity of each band from Spt2p was divided by the intensity of band from tubulin control. The number generated from time 0 was set to 1 and the fold induction was calculated. B) Chronic expression of Spt2p causes growth arrest. (0.39 MB TIF) [file pgen.1000290.s001.tif]
