# Supplementary material for: Spt2p Defines a New Transcription-Dependent Gross Chromosomal Rearrangement Pathway
Source: PLoS Genet. 2008 Dec 5;4(12):e1000290. doi: 10.1371/journal.pgen.1000290 (PMC2585797; doi:10.1371/journal.pgen.1000290)
Supplement: Table S1 — S. cerevisiae strains used in this study. (0.16 MB DOC) [file pgen.1000290.s002.doc]

Table S1. *S. cerevisiae* strains used in this study

| Strains | Relevant genotype | Plasmid |
| --- | --- | --- |
| YKJM2636 | *spt2::TRP1* |  |
| YKJM2233 | *bre1::His3* |  |
| YKJM4415 | *rad6::His3* |  |
| YKJM4459 | *bur2::His3* |  |
| YKJM1445 | *cdc73::KAN* |  |
| YKJM4177 | *hir1::His3* |  |
| YKJM4503 | *swr1::His3* |  |
| YKJM934 | *dot1::His3* |  |
| YKJM4501 | *set1::His3* |  |
| YKJM4505 | *dst1::His3* |  |
| YKJM4473 | *rnh1::His3* |  |
| YKJM4475 | *rnh201::His3* |  |
| YKJM4531 | *fir1::His3* |  |
| YKJM4563 | *hpr1::His3* |  |
| YKJM2416 | *Wild type* | pYES3CT |
| YKJM2472 | *Wild type* | pKJM378(Spt2p) |
| YKJM4820 | *Wild type* | pKJM989 |
| YKJM4576 | *Wild type* | pKJM924 (1-96) |
| YKJM4578 | *Wild type* | pKJM926 (100-162) |
| YKJM4580 | *Wild type* | pKJM928 (224-304) |
| YKJM4582 | *Wild type* | pKJM930 (304-333) |
| YKJM4706 | *Wild type* | pKJM985 (K325A) |
| YKJM4611 | *yku80::His3* | pYES3CT |
| YKJM4613 | *yku80::His3* | pKJM378 |
| YKJM4877 | *yku80::His3* | pKJM989 |
| YKJM4584 | *rad1::His3* | pYES3CT |
| YKJM4586 | *rad1::His3* | pKJM378 |
| YKJM4881 | *rad1::His3* | pKJM989 |
| YKJM4588 | *rad10::His3* | pYES3CT |
| YKJM4590 | *rad10::His3* | pKJM378 |
| YKJM4879 | *rad10::His3* | pKJM989 |
| YKJM4607 | *tlc1::His3* | pYES3CT |
| YKJM4609 | *tlc1::His3* | pKJM378 |
| YKJM4883 | *tlc1::His3* | pKJM989 |
| YKJM4425 | *bre1::His3* | pYES3CT |
| YKJM4427 | *bre1::His3* | pKJM378 |
| YKJM4818 | *bre1::His3* | pKJM989 |
| YKJM4461 | *rad6::His3* | pYES3CT |
| YKJM4463 | *rad6::His3* | pKJM378 |
| YKJM4857 | *rad6::His3* | pKJM989 |
| YKJM4465 | *bur2::His3* | pYES3CT |
| YKJM4467 | *bur2::His3* | pKJM378 |
| YKJM4859 | *bur2::His3* | pKJM989 |
| YKJM4478 | *dot1::His3* | pYES3CT |
| YKJM4480 | *dot1::His3* | pKJM378 |
| YKJM4816 | *dot1::His3* | pKJM989 |
| YKJM5094 | *set1::His3* | pYES3CT |
| YKJM4519 | *set1::His3* | pKJM378 |
| YKJM4861 | *set1::His3* | pKJM989 |
| YKJM4162 | *cdc73::KAN* | pYES3CT |
| YKJM4164 | *cdc73::KAN* | pKJM378 |
| YKJM4855 | *cdc73::KAN* | pKJM989 |
| YKJM4206 | *hir1::His3* | pYES3CT |
| YKJM4208 | *hir1::His3* | pKJM378 |
| YKJM4814 | *hir1::His3* | pKJM989 |
| YKJM5096 | *swr1::His3* | pYES3CT |
| YKJM4517 | *swr1::His3* | pKJM378 |
| YKJM4863 | *swr1::His3* | pKJM989 |
| YKJM4521 | *dst1::His3* | pKJM378 |
| YKJM4529 | *dst1::His3* | pYES3CT |
| YKJM4865 | *dst1::His3* | pKJM989 |
| YKJM4573 | *hpr1::His3* | pYES3CT |
| YKJM4575 | *hpr1::His3* | pKJM378 |
| YKJM4822 | *hpr1::His3* | pKJM989 |
| YKJM4559 | *fir1::His3* | pYES3CT |
| YKJM4561 | *fir1::His3* | pKJM378 |
| YKJM4867 | *fir1::His3* | pKJM989 |
| YKJM4166 | *Wild type* | pYES3CT, pYX243 |
| YKJM4168 | *Wild type* | pKJM378, pYX243 |
| YKJM4752 | *Wild type* | pKJM378, pKJM1011 (RNH1) |
| YKJM5053 | *ung1* | pKJM989 |
| YKJM5088 | *Wild type TEF-TRP1 (CEN to TEL)* |  |
| YKJM5090 | *Wild type TEF-TRP1 (TEL to CEN)* |  |
| YKJM5098 | *Wild type* | pESC |
| YKJM5100 | *Wild type* | pESC-AID |
| YKJM5222 | *Wild type Gal-TRP1 (TEL to CEN)* |  |

All strains are isogenic to Winston S288w background, RDKY3615 [*ura3-52, leu2∆1, trp1∆63, his3∆200, lys2∆Bgl, hom3-10, ade2∆1, ade8, YEL069::URA*] for general GCR assay except for the mutations and plasmids indicated. pKJM378 and pKJM989 are plasmids expressing Spt2p under the galactose promoter in the pYES3CT (TRP1) backbone. pKJM989 has a FLAG tag in the N-terminus. pKJM1011 is a plasmid expressing RNase H under the galactose promoter in the pYX243 backbone (LEU2). pKJM924, pKJM926, pKJM928, and pKJM930 express different fragments of Spt2p under the galactose promoter in pYEST3CT backbone. The number in parentheses indicates the amino acids expressed by each plamid. pKJM985 expresses the K325A mutated Spt2p. pKJM1011 is a plasmid expressing RNase H under the galactose promoter in the pYX243 (LEU2) backbone. pESC-AID expresses human AID under the galactose promoter in the pESC (LEU2) backbone. All strains were generated in this study except YKJM2233 that was made previously [1].

**References**

1. Motegi A, Kuntz K, Majeed A, Smith S, Myung K (2006) Regulation of gross chromosomal rearrangements by ubiquitin and SUMO ligases i*n Saccharomyces cerevisi*ae. Mol Cell Biol 26: 1424-1433.
